# The Physical Role of Mesenchymal Cells Driven by the Actin Cytoskeleton Is Essential for the Orientation of Collagen Fibrils in Zebrafish Fins

**DOI:** 10.3389/fcell.2020.580520

**Published:** 2020-10-14

**Authors:** Junpei Kuroda, Takeshi Itabashi, Atsuko H. Iwane, Toshihiro Aramaki, Shigeru Kondo

**Affiliations:** ^1^Graduate School of Frontier Bioscience, Osaka University, Suita, Japan; ^2^RIKEN Center for Biosystems Dynamics Research, Higashi-Hiroshima, Japan

**Keywords:** collagen, mesenchymal cell, extracellular matix, zebrafish, fin, actin, FIB-SEM, 3D structural model

## Abstract

Fibrous collagen imparts physical strength and flexibility to tissues by forming huge complexes. The density and orientation of collagen fibers must be correctly specified for the optimal physical property of the collagen complex. However, little is known about its underlying cellular mechanisms. Actinotrichia are collagen fibers aligned at the fin-tip of bony fish and are easily visible under the microscope due to their thick, linear structure. We used the actinotrichia as a model system to investigate how cells manipulate collagen fibers. The 3D image obtained by focused ion beam scanning electron microscopy (FIB-SEM) showed that the pseudopodia of mesenchymal cells encircle the multiple actinotrichia. We then co-incubated the mesenchymal cells and actinotrichia *in vitro*, and time-lapse analysis revealed how cells use pseudopods to align collagen fiber orientation. This *in vitro* behavior is dependent on actin polymerization in mesenchymal cells. Inhibition of actin polymerization in mesenchymal cells results in mis-orientation of actinotrichia in the fin. These results reveal how mesenchymal cells are involved in fin formation and have important implications for the physical interaction between cells and collagen fibers.

## Introduction

Collagens are a major component of the extracellular matrix (ECM) that provides physical strength to connective tissues (Sherman et al., 2015). Collagen proteins form polymers with complex structures, which exhibit different physical properties depending on their configuration (Hulmes, 2002; Kadler et al., 2007). It is important to understand the principles that determine the structure of collagen complexes, because abnormalities in the density, thickness, and orientation of collagen fibers are known to cause a variety of diseases (Kadler et al., 2007).

The structure of collagen polymers is considered to be fundamentally determined by the self-assembling properties of the molecules themselves (Mouw et al., 2014). In vertebrates, there are 28 types of collagen superfamilies, each of which is polymerized in a different manner (Kadler et al., 2007; Ricard-Blum, 2011; Mouw et al., 2014). For example, fibrous collagen with linear polymers, such as type 1 and type 2 collagen, is abundant in bones, cartilage, tendons, and skin (Hulmes, 2002; Fang and Holl, 2013). In contrast, type 4 collagen, which is contained in vascular epithelium, and others, creates a network structure in the basement membrane (Fidler et al., 2017). Some previous studies have suggested that the self-assembling activity of collagen molecules is insufficient for the formation of precise complexes. For example, several ECM factors that co-assemble with collagen fibers to regulate density and thickness have been reported (Danielson et al., 1997; Zhang et al., 2009; Goodyear et al., 2017). Cells in connective tissues are also suggested to interact with collagen fibers to change their orientation (Jansen et al., 2013; Shi et al., 2014; Coelho et al., 2017; Kim et al., 2017; Piotrowski-Daspit et al., 2017; Xie et al., 2017). However, due to their deep location in the body and the difficulty in observing the fibers themselves under living conditions, the mechanisms determining the density, bundle size, and orientation of collagen fibers remain largely unknown.

The fin tips of fish are regularly lined with bundles of acicular collagen fibers called actinotrichia. Individual actinotrichia primarily consist of type1 and type2 collagen and Actinodin (And) proteins (Zhang et al., 2010; Durán et al., 2011; König et al., 2018). They are structured linearly, unbranched, and arranged radially in an orderly fashion at the growth end of the fins (Akimenko et al., 2003; Pfefferli and Jaźwińska, 2015). During the fin regeneration process, actinotrichia are rapidly produced and support the soft fin tissue composed of epidermis and mesenchyme (Mari-Beffa et al., 1989; Santamaría and Becerra, 1991; Akimenko et al., 2003; LeBert et al., 2015, 2018; Pfefferli and Jaźwińska, 2015; Thorimbert et al., 2015; König et al., 2018). Actinotrichia are considered essential for fin morphogenesis because the morphology of fins and fin bones is abnormal in some mutants with abnormal size and arrangement of actinotrichia (Huang et al., 2009; Durán et al., 2011; Lalonde and Akimenko, 2018). Moreover, as fins are a flat, thin structure, individual actinotrichia can be easily observed under the microscope.

Extensive molecular genetic studies on the formation and function of actinotrichia have been conducted in zebrafish. Several studies using transmission electron microscopy (TEM) have shown that actinotrichia are distributed between the basement membrane just below the basal epithelial cells and mesenchymal cells (Wood and Thorogood, 1987; Böckelmann and Bechara, 2010). In zebrafish larva with Col1a1a and Col2a1b function inhibited using morpholino antisense oligos, actinotrichia production is inhibited and the fin fold growth is defective (Durán et al., 2011). A *col1a1a* mutant showed an abnormally thickened actinotrichia and a decrease in their number (Durán et al., 2011). In addition, the fins shrunk and the fin bones showed a wavy shape in this mutant (Durán et al., 2011). In the mutant of *prp* that encodes type IX collagen, actinotrichia orientation is disordered, which results in thickened fins and wavy fin bone (Huang et al., 2009). These studies suggest that proper density and orientation of actinotrichia are necessary for generating fins with the correct morphology. Recently, specific ablation of mesenchymal cells in fins has been shown to disrupt the orientation of actinotrichia (Lalonde and Akimenko, 2018). This suggests that fin mesenchymal cells may be involved in the formation of the orientation of actinotrichia (Lalonde and Akimenko, 2018).

Genetic methods are quite effective to estimate the contribution of certain cells to the formation and maintenance of actinotrichia, but it is difficult to rule out the contribution of environmental factors and cells located in the vicinity. Further, when the function of a gene is lost in a particular cell, the field itself changes, affecting other cells as well, which makes it difficult to isolate the function of a single cell type. If the interaction between purified cells and actinotrichia could be observed *in vitro*, it would complement the weaknesses of *in vivo* experiments. As a first example of such an experiment, *in vitro* culture of basal keratinocytes purified from fins has shown that actinotrichia formation begins intracellularly (Kuroda et al., 2018).

In this study, we aimed to investigate the interaction between cells and collagen fibers by studying bundle formation of actinotrichia and their orientation as a model system. First, to understand the physical relationship between actinotrichia and surrounding cells, we performed the FIB-SEM analysis, and found that fin mesenchymal cells hold multiple actinotrichia using their pseudopodia. Next, we purified mesenchymal cells and actinotrichia from fins and cultured them *in vitro*, observing that mesenchymal cells hold multiple actinotrichia by the pseudopodia and bundle them together to align their orientations. Furthermore, the *in vitro* phenomenon was related to the actual, actinotrichia alignment in fins, as the inhibition of actin polymerization in mesenchymal cells resulted in abnormal actinotrichia orientation in the fins. These results elucidate the function of mesenchymal cells in fin formation and provide a new way to study the interaction between cells and collagen fibers.

## Materials and Methods

### Zebrafish Lines

Animal experiments were approved by the animal care and use at Osaka University. Zebrafish were maintained under the standard laboratory conditions and were treated as previously described (Westerfield, 1995). We used the AB strains as wild type zebrafish lines. Strains were generated for this study:

*Tg [5xand1(MC): Lifeact-mCherry]*, *Tg [5xand1(2p): And1^full^-GFP]*, *Tg (and1 1.4k: H2B-mRFP)*, *Tg (and1 1.4k: And1^480bp^-KikGR)*, *Tg [5xand1(MC): RhoA^WT^: Ires: H2B-mRFP]*, and *Tg [5xand1(MC): RhoA^T19N^: Ires: H2B-mRFP]*.

These lines were created by injecting the tol2 plasmid with Tol2 transposase (Urasaki et al., 2006) into AB embryos. All zebrafish used in experiments were healthy and in normal immune status, not involved in previous procedures and drug treatment naive.

### Microscopy for the Fluorescent Imaging

Live zebrafish larvae were anesthetized in tricaine (MS-222) and their median fin folds were fixed with 4% paraformaldehyde (PFA) in PBS O/N at 4°C. The images of fins were obtained using a BZ-X710 (Keyence) with 10× NA 0.45 PlanApo and 20× NA 0.45 PlanFluor objective (Nikon) and LSM 780 (Carl Zeiss) with 63× NA 1.40 Oil PlanApo objective (Carl Zeiss). Images of the cultured mesenchymal cells were acquired using LSM 780 (Carl Zeiss) with 63× NA 1.40 Oil PlanApo objective (Carl Zeiss). ZEN (Carl Zeiss) and FIJI were used as image software for *z* projections. Live cell imaging was performed on a LSM780 (Carl Zeiss) with 20× NA 0.8 PlanApo (Carl Zeiss). The green and red fluorescence signal was detected by GaAsP detectors with 488 and 561 nm laser for live cell imaging. The images were processed into videos using Imaris software (Bitplane).

### Three-Dimensional Ultra-Structural Analysis Using FIB-SEM

The larval median fin folds were fixed in 2% glutaraldehyde (GA) and 2% PFA in PBS at 4°C overnight. They were post-fixed with 2% osmium tetroxide in PBS for 30 min at room temperature. After post-fixation, they were stained with 1% uranyl acetate in distilled water for 2 h at room temperature and then, with 0.02 M lead nitrate in 0.03 M L-aspartic acid solution for 30 min at 60°C. Samples were dehydrated in increasing concentrations of Ethanol (50, 70, 90, 95, and 100%), embedded in the Epoxy-resin mixture containing TAAB Epon812, MNA, DDSA and DMP30 and polymerized in an oven for 4 days at increasing stepwise temperature.

The sample blocks were mounted on the metal SEM stub with a conductive carbon cement (Agar Scientific, Stansted, United Kingdom). The blocks were trimmed with a glass knife to expose the region of interest, and then coated with carbon using a Q150T coater (Quorum Technologies, Laughton, United Kingdom) and osmium using a Neoc coater (Meiwafosis, Tokyo, Japan) before loading into the FIB-SEM.

FIB-SEM tomography was performed with a FEI Helios G4 UC dual beam system (Thermo Fisher Scientific, Waltham, MA, United States). To enable stable image acquisition over a long period of time, a thin platinum layer (1 μm thick) was deposited on the top of the region of interest. Then, the cross-sectional imaging face was obtained by milling a trench in front of the targeted area. To acquire 3D volume images, Auto Slice and View Ver. 4.2 software (Thermo Fisher Scientific, Waltham, MA, United States) was used for sequential FIB milling and SEM imaging. Serial milling at 15 nm (Figure 2) intervals was obtained at an acceleration voltage of 30 kV and a current of 0.24 nA. Subsequent SEM imaging of the block face using the in-column BSE detector was performed at an acceleration voltage of 2 kV, a current of 0.2 nA, 4 mm working distance and 10 μs dwell time. For image analysis, serial images obtained with the voxel size of 8 nm × 10 nm × 15 nm in Figure 2 were first aligned, and then cropped, median filtered, reconstructed and manually segmented using the Amira ver. 2019.4 software (Thermo Fisher Scientific, Waltham, MA, United States).

### *In vitro* Culture of Fin Mesenchymal Cells With Actinotrichia

Mesenchymal cells were harvested from the median fin folds of 3 dpf larvae. Zebrafish larvae were anesthetized in a standard tricaine (MS-222) solution (0.4% in breeding water) and median fin folds were dissected. The dissected fins were treated with trypsin solution [2.5 mg/mL trypsin (TRL; Worthington), 1.0 mg/mL BSA (Sigma-Aldrich), 1 mM EDTA in PBS] for 10 min at 28 °C. After trypsin treatment, the fins were shaken with PBS for 5 min at 1,000 rpm at 28°C (repeat shaking at three times) and the cell suspension was recovered into one centrifugation tube. Subsequently, the samples were centrifuged at 100 G for 10 min, and the pellet was resuspended in L15 (Gibco) medium with 10% FBS (Gibco) and spread onto a glass bottom dish. Matrigel, type I collagen, type IV collagen were used for the coating substrates on the dish. Matrigel (Corning) was diluted to 1/50 with PBS and coated on the dish. Type 1 collagen (Nitta Gelatin) and type IV collagen (Corning) was diluted to 1/10 with 0.05 N HCl and coated on the dish. Actinotrichia were isolated from the median fin folds of larvae by the above-mentioned method and corrected in culture dishes. The cultured cells and actinotrichia samples were incubated at 28°C and L15 medium with 10% FBS was changed to a fresh one every day.

### Scanning Electron Microscopy

Cultured mesenchymal cells derived from fins of *Tg[5xand1(MC): Lifeact-mCherry]* were fixed with 2% PFA and 2% GA in PBS O/N at 4°C. After fixation, samples were dehydrated in ethanol solutions of increasing concentration (25, 50, 70, 80, 90, 95, and 100%) and then they were frozen in t-butyl alcohol at −30°C overnight. Next day, frozen t-butyl alcohol was sublimated in the vacuum evaporator and finally dried samples were sputter coated with gold particles. Images were acquired on a HITACHI S-4800 scanning electron microscope.

### Photo-Conversion of Actinotrichia

Actinotrichia in the larval fin of *Tg (1.4k and1: And1^480bp^-KikGR)* were used for photo-conversion experiments. The samples were exposed to UV light (365 nm) for 5 s to 2 min using LED-EXTA (OptoCode) with EX-365 (OptoCode). Their fluorescent colors were confirmed by a BZ-X710 microscopy (Keyence) with 10× NA 0.45 PlanApo and 20× NA 0.45 PlanFluor objective (Nikon). The isolated actinotrichia were spread onto a grid glass (Matsunami) coated with Matrigel and used for the culture experiment.

### Staining of Nucleus and Actin Cytoskeleton

The cultured basal keratinocytes and mesenchymal cells were incubated with Syto 9 (Thermo Fisher; 1:1000) in L15 medium for 30 min at 28°C. After the incubation, they were washed with PBS and re-cultured in L15 medium (Gibco) with 10% FBS (Gibco) at 28°C. For the observation of actin cytoskeleton, the cultured mesenchymal cells were fixed with 2% PFA in PBS O/N at 4°C. After the fixation, the samples were washed with PBS and incubated with Alexa Fluor 654 conjugated phalloidin (Invitrogen; 1:200) in PBS O/N at 4°C. Subsequently, the samples were wash with PBS and used for confocal imaging.

### Drug Treatment of the Cultured Cells

Harvested mesenchymal cells and basal keratinocytes from larval fins at 3 dpf were treated with G418 (Nacalai) and NaN_3_ (Wako) in the culture experiment. G418 was adjusted to a concentration of 200 μg/ml in L15 medium and NaN_3_ was adjusted to a concentration of 0.1% in L15 medium. To disturb the cellular actomyosin in the culture experiment, harvested mesenchymal cells were treated with 1.0 μg/ml Cytochalasin D (CytoD) (Sigma-Aldrich), 2.0 μg/ml CT04 (Cytoskeleton), 50 μM Blebbistatin (Sigma-Aldrich) and 0.1% DMSO.

### Immunofluorescence Labeling

The cultured mesenchymal cells derived from the fins of *Tg[5xand1(MC): Lifeact-mCherry]* were fixed with 2% PFA in PBS O/N at 4°C. The fixed samples were washed in PBS and permeabilized for 5 min with 0.2% tween 20 in PBS. Subsequently the samples were blocked with 1% BSA in PBS for 1 h at room temperature, and incubated with primary antibodies in PBS O/N at 4°C. After the incubation with primary antibodies, the samples were washed with PBS and incubated with secondary antibodies in PBS for 2 h at room temperature. After subsequent washing with PBS, the samples were used for the confocal imaging. Rabbit anti-beta Tubulin antibody conjugated with Alexa Fluor 488 (Cell Signaling; 1:100) and rabbit anti-pFAK antibody (GeneTex; 1:100) were used as primary antibodies. Alexa Fluor 488 goat anti-rabbit IgG antibody (Invitrogen; 1:200) was used as secondary antibody to detect pFAK.

### Measurement of the Nucleus of Mesenchymal Cells and Statistical Analysis

Elongation ratio and direction of the nucleus of mesenchymal cells at ventral-mid area (90 μm × 90 μm) in median fins expressing *RhoA*^WT^ and *RhoA*^T19N^ were measured and analyzed with FIJI software. Excel (Microsoft) and R (R Development Core Team) were used for drawing graphs and statistical analyses. For statistical analyses, the data were examined using an unpaired *t*-test. *P*-values are summarized as ^∗∗∗^*p* < 0.0001.

## Results

### Orientation of Actinotrichia and the Surrounding Cells

Previous studies have suggested that actinotrichia are distributed between basal keratinocytes and mesenchymal cells in fins (Wood and Thorogood, 1987; Smith et al., 2008; König et al., 2018; Kuroda et al., 2018). Basal keratinocytes are in contact with the outer part of the two actinotrichia rows whereas mesenchymal cells are in contact with the inner part. To determine which (or both) cells contribute to the correct orientation of actinotrichia, it is important to determine the orientation of each cell at the fins. Therefore, we investigated the relationship between cellular and actinotrichia orientation by imaging these two cell types.

**FIGURE 1:**
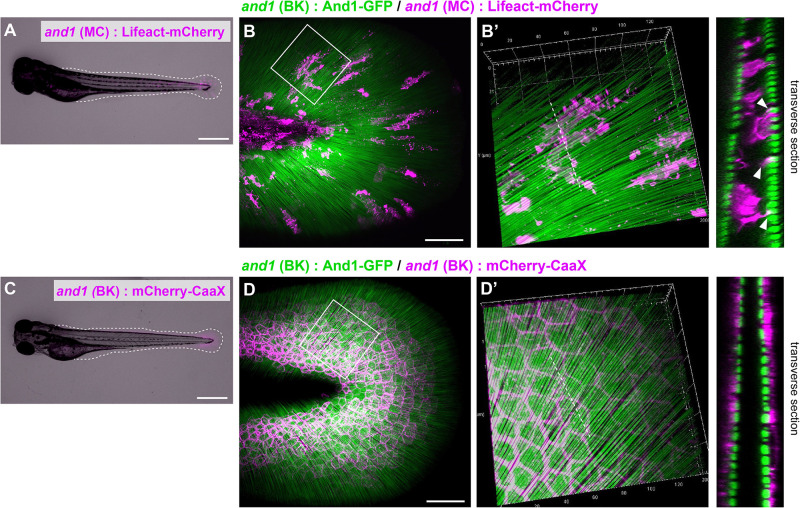
Distribution pattern of the mesenchymal cells in larval fins. **(A)** Image of the whole body and **(B)** the median fin fold of the transgenic (TG) larva expressing Lifeact-mCherry under the 5^x^*and1*(MC) promoter. The white dot line shows the outline of median fin fold. **(B)** Localization pattern of mesenchymal cells (expressing Lifeact-mCherry: magenta) and actinotrichia (labeled by And1-GFP: green) in the larval fin. **(B′)** Magnified image of the white box in **(B)**. Transverse section image of the white dotted line is shown in the right panel. Actin-rich pseudopodia interact with actinotrichia (white arrowheads). **(C)** Image of the whole body and **(D)** the median fin fold of TG larva expressing mCherry-CaaX under the *and1*(BK) promoter. The white dotted line shows the outline of the median fin fold. **(D)** Localization pattern of basal keratinocytes (expressing mCherry-CaaX: magenta) and actinotrichia (labeled by And1-GFP: green) in the larval fin. **(D′)** The magnified image of white box in **(D)**. Transverse section image at the white dotted line is shown in the right panel. All larvae were at the stage of 3 dpf. MC, mesenchymal cell specific promoter designated as 2PΔepi in the previous paper (Lalonde et al., 2016); BK, basal keratinocyte specific promoter designated as 1.4 k *and1* pro in the previous paper (Kuroda et al., 2018). Scale bars: 500 μm in **(A,C)**, 50 μm in **(B,D)**.

First, we visualized each cells by expressing mCherry under the control of a cell-specific promoter (Lalonde et al., 2016; Kuroda et al., 2018; Phan et al., 2019). Actinotrichia were also simultaneously visualized by expressing And1-GFP under the control of *and1* promoter, as we reported previously (Kuroda et al., 2018). Figures 1A,C show the larva used for the observation and Figures 1B,D show the fin fold. Because the F1 fish of 5x*and1* (MC): Lifeact-mCherry for the mesenchymal cells labeling have more cell overlap and individual cell morphology was difficult to distinguish, we observed the mosaic of expression in F0 fish. In F0 fish expressing Lifeact-mCherry with the fin mesenchymal cell-specific promoter [5x*and1* (MC)], fluorescence was distributed only inside the actinotrichia layer, indicating the correct labeling of mesenchymal cells (Figure 1B′). They also appeared to interact with actinotrichia by extending actin-rich pseudopodia (Figures 1B,B′). The morphology of each mesenchymal cell was elongated and oriented radially along the actinotrichia (Figures 1B,B′). We selected Lifeact-mCherry as the label protein for visualizing mesenchymal cells in this study because the pseudopodia of mesenchymal cells could not be visualized clearly in fish expressing mCherry-CaaX using the same promoter (data not shown). In fish expressing mCherry-CaaX with a basal keratinocyte-specific promoter, the fluorescence was observed outside the actinotrichia layer, indicating that the basal keratinocytes were labeled correctly (Figure 1D′). Basal keratinocytes showed a polygonal shape and were attached to neighboring cells without gaps (Figures 1D,D′). This indicated that they had no correlation with the orientation of the actinotrichia. These results suggest that mesenchymal cells distributed in the inner layer, rather than the outer basal keratinocytes, may be involved in actinotrichia orientation.

**FIGURE 2:**
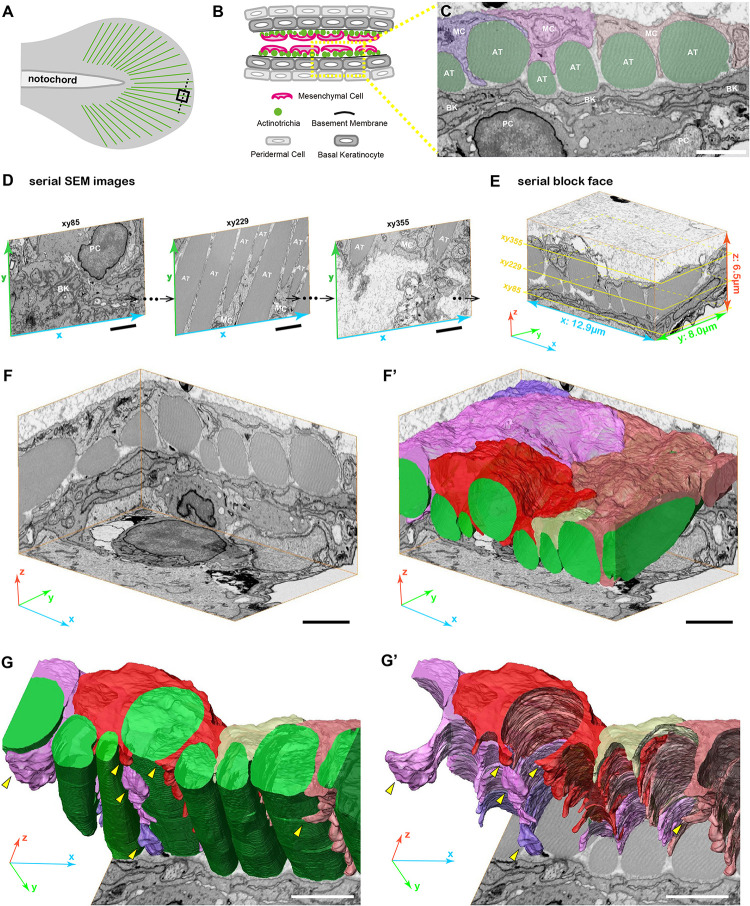
3D reconstruction of fin mesenchymal cells and actinotrichia by FIB-SEM analysis. **(A)** Illustration of the 3 dpf larval median fin. Black boxed region (15 μm × 15 μm) in the fin tip was used for the FIB-SEM analysis. **(B)** Schematic diagram of the transverse section of the black dotted line in **(A)** at the fin tip. Actinotrichia are distributed inside the basement membrane. **(C)** An example of SEM image of the transverse section at the fin tip. Mesenchymal cells (shown in purple, pink, and orange color) develop the long pseudopodia structures and directly interact with actinotrichia (shown in green color). **(D)** Serial SEM images at *x*-*y*- plane obtained by FIB-SEM observation. **(E)** Three-dimensional view of the serial block surface image, 12.9 μm × 8.0 μm × 6.5 μm. The positions of each SEM images in **(D)** are shown in the yellow boxes. **(F)** Three-dimensional view of the reconstructed sequential SEM images and **(F′)** the 3D reconstruction of eight actinotrichia (labeled by green color) and five mesenchymal cell domains (labeled by purple, pink, red, yellow, and orange color). **(G)** 3D reconstruction of actinotrichia and mesenchymal cell domains and **(G′)** 3D reconstruction of mesenchymal cell domains viewed from the epidermal cell layer. Multiple pseudopodia structures are developed from each mesenchymal cell domains and hold actinotrichia fibrils (yellow arrowheads). MC, mesenchymal cell; AT, actinotrichia; BK, basal keratinocyte; PC, peridermal cell. Scale bars: 2 μm.

### 3D Morphological Interaction Between Mesenchymal Cell and Actinotrichia *in vivo*

Next, we examined the 3D morphology around actinotrichia in the tip region of the larval fin fold (Figure 2A) using focused ion beam scanning electron microscopy (FIB-SEM) to elucidate the morphological relationship between mesenchymal cells and actinotrichia in more detail. For the FIB-SEM experiment, we analyzed a partial area in one actinotrichia layer (Figures 2B,C). Using the continuous SEM images obtained via 15 nm intervals (Figure 2D and Supplementary Movies 1–3) we produced a Serial Block Face (SBF: *x*, *y*, *z*, 12.9, 8.0, and 6.5 μm) with a software (Amira ver. 2019.4) (Figure 2E). Then, sub SBF was cropped as shown the illustration (Figure 2F). Subsequently, we extracted the contours of actinotrichia and mesenchymal cells in each SEM images and generated 3D structural model (Figure 2F′). As shown in Figure 1, it is mesenchymal cells that interact more strongly with actinotrichia. The 3D reconstruction of the extracted image shows that five mesenchymal cell domains hold actinotrichia fibrils with long pseudopodia structures (Figures 2G,G′ and Supplementary Movie 4). The multiple pseudopodia structures developed from each cell domains invade into the space between actinotrichia and directly contact with the surface of the fibrils (Figures 2G,G′ and Supplementary Movie 4). In addition, some pseudopodia extend to deeper region and surround the fibrils (yellow arrowheads in Figures 2G,G′). These characteristic morphologies suggest that mesenchymal cells are strongly involved in the orderly alignment formation of actinotrichia.

### *In vitro* Interaction Between Mesenchymal Cell and Actinotrichia

**FIGURE 3:**
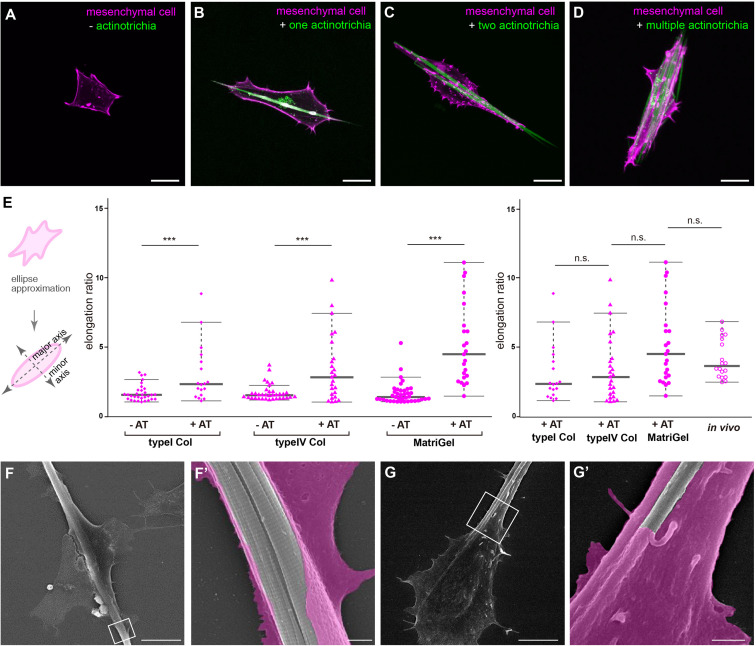
Interaction between mesenchymal cells and actinotrichia *in vitro.* **(A–D)** The morphology of primary cultured mesenchymal cells at day 2 after culture on a Matrigel-coated dish. Mesenchymal cells and actinotrichia were isolated from TG larval fins [TG; 5^x^*and1*(MC]: Lifeact-mCherry/5^x^*and1*(2P): And1^full^-GFP). **(A)** In the condition without actinotrichia, mesenchymal cells showed the symmetric morphology. **(B–D)** In the condition with actinotrichia, mesenchymal cells elongated along a single actinotrichia **(B)**, two actinotrichia **(C)** and multiple actinotrichia **(D)**. **(E)** Cell aspect ratio of mesenchymal cells. Mesenchymal cells in contact with actinotrichia had a more elongated shape compared to the cells not in contact with actinotrichia. **(F–G′)** SEM images of cultured mesenchymal cells holding the actinotrichia on a Matrigel-coated dish. The actinotrichia was surrounded with the plasma membrane of the mesenchymal cell **(F)** and partially embedded inside the mesenchymal cell **(G)**. The magnified images in white box are shown. *P*-values: ***P < 0.0001. AT, actinotrichia. Scale bars: 20 μm in **(A–D)**, 10 μm in **(F,G)**, 1 μm in **(F′,G′)**.

To elucidate the *in vivo* interaction of mesenchymal cells with actinotrichia, we investigated their physical interaction *in vitro* by performing a primary culture. Prior to conducting the *in vitro* study, we established a new actinotrichia-visualized zebrafish line [5x*and1* (2k): And1^full^-GFP] to allow more detailed observation of the cell and actinotrichia dynamics (Supplementary Figure 1). The fins of this new actinotrichia-visualized zebrafish showed even greater fluorescence intensity of the actinotrichia compared to the previously reported visualization line (Kuroda et al., 2018) (Supplementary Figures 1, 2). Using previously reported methods (Kuroda et al., 2018), we first isolated the actinotrichia and mesenchymal cells from the larval fins of our new transgenic F1 line [5x*and1* (2k): And1^full^-GFP/5x*and1* (MC): Lifeact-mCherry] and cultured them in culture dishes coated with various kinds of substrates. In the absence of contact with actinotrichia, mesenchymal cells were not particularly directional and were spread out on Matrigel-coated dishes (Figure 3A). Interestingly, upon contact with actinotrichia, mesenchymal cells were elongated along their long axis, similar to the *in vivo* observations (Figures 3B–D and Supplementary Movies 5–7). There was a significant difference in the elongation ratio of mesenchymal cells in contact with and without actinotrichia (Figure 3E). Furthermore, the *in vitro* and *in vivo* values were found to be nearly equal (Figure 3E). The substrate coating the dish did not seem to have much of an effect and a positive interaction between mesenchymal cells and actinotrichia was observed regardless of the coating substrate (Figure 3E and Supplementary Movies 5–7). Mesenchymal cells showed the propensity to elongate along their long axis when in contact with actinotrichia on all substrates (Figure 3E). Therefore, in subsequent experiments, we used Matrigel, which is closest to the components of the basement membrane, as the coating substrate. Next, we performed SEM observations to investigate the detailed structure of the mesenchymal cell membrane in contact with actinotrichia. In many cases, the mesenchymal cells developed a thin membrane-like protrusion that enveloped the actinotrichia (Figures 3F,F′). In some cases, we observed that actinotrichia partially penetrated the interior of the mesenchymal cells (Figures 3G,G′). These images suggest that the membranes of mesenchymal cells have a strong affinity for actinotrichia. The above results indicate that mesenchymal cells have the property of interacting with actinotrichia and elongating their morphology both *in vitro* and *in vivo*.

### Live Cell Imaging of Mesenchymal Cells During Their *in vitro* Alignment With Actinotrichia

**FIGURE 4:**
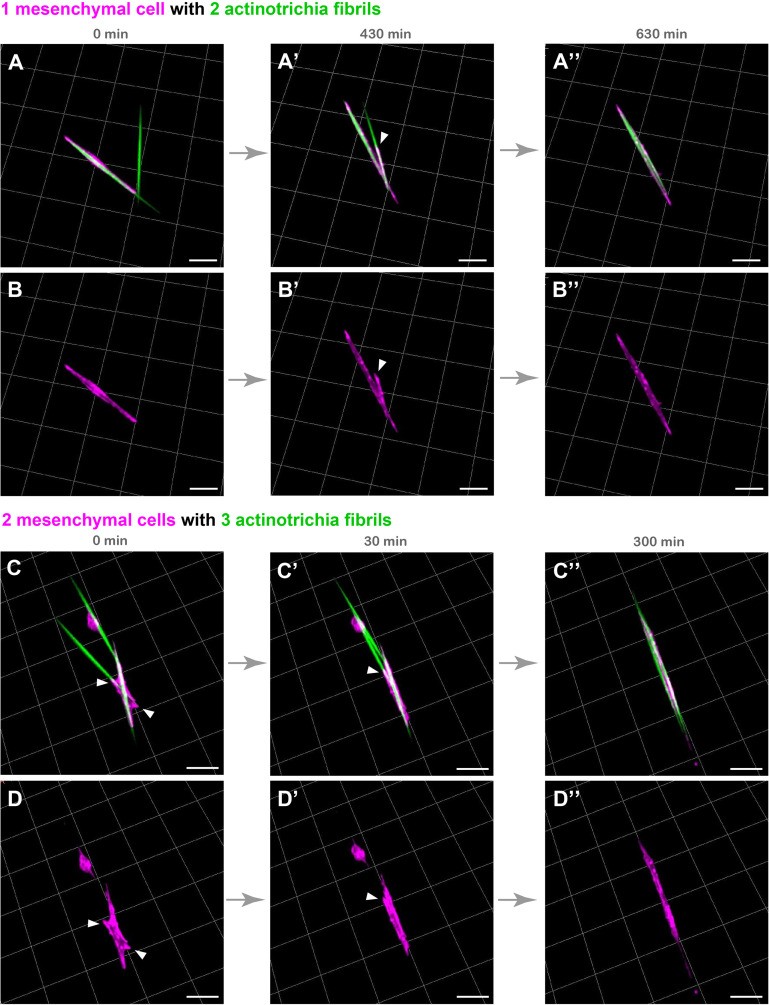
Live cell imaging of cultured mesenchymal cells holding the actinotrichia fibrils. Captured time-lapse images for the interaction between mesenchymal cells and actinotrichia fibrils. Mesenchymal cells and actinotrichia were isolated from the fins of F1 TG larvae [TG; 5^x^*and1*(MC): Lifeact-mCherry/5^x^*and1*(2P): And1^full^-GFP] and cultured on a Matrigel-coated dish. **(A)** A single mesenchymal cell initially surrounded one of two different actinotrichia fibrils. **(A′)** At 430 min, the mesenchymal cell started to develop a filopodia like structure (white arrowheads) and to hold another fibril. Finally, **(A′′)** at 630 min, two fibrils were completely aligned in the direction of the longitudinal axis of the mesenchymal cell. The fluorescence images of only a mesenchymal cell are shown in the bottom panels **(B–B′′)**. **(C)** Two mesenchymal cells initially developed their pseudopodia (white arrowheads) and attached to three different fibrils, and **(C′)** gradually changed their morphology and moved the fibrils. **(C′′)** At 300 min, the two cells extended in the same direction and all of three fibrils were oriented along the longitudinal axis of the two mesenchymal cells. The fluorescence images of only two mesenchymal cells are shown in bottom panels **(D–D′′)**. Scale bars: 20 μm.

Although the above experimental results indicate that mesenchymal cells have the property to hold multiple actinotrichia, the characteristic behavior of these cells may consequently contribute to align the orientation of actinotrichia. In order to investigate this, we spread actinotrichia and mesenchymal cells on culture dishes coated with Matrigel. We then searched for mesenchymal cells in close proximity to multiple unaligned actinotrichia and observed their dynamics by live imaging. Interestingly, a single mesenchymal cell was observed to physically move two differently oriented actinotrichia and align their orientations (Figures 4A–B′′ and Supplementary Movie 8). In the beginning, two actinotrichia fibrils intersected at an angle of about 60 degrees, and the mesenchymal cell surrounded the left one of them (Figures 4A,B and Supplementary Movie 8). Subsequently, the mesenchymal cells extended their pseudopods toward the right fibril, and the angle between the two fibrils gradually decreased (Figures 4A′,B′ and Supplementary Movie 8). Eventually, the two fibrils were aligned and placed parallel to each other (Figures 4A′′–B′′ and Supplementary Movie 8). We also observed that two different mesenchymal cells moved three actinotrichia fibrils, which were oriented in different directions, to align their orientations (Figures 4C–D′′ and Supplementary Movie 9). From the intersection of each fibril, the mesenchymal cells extended to the three fibrils, and finally all the fibrils were aligned (Figure 4C–D′′ and Supplementary Movie 9). These results suggest that mesenchymal cells are capable of aligning the orientation of actinotrichia independently.

### Mesenchymal Cell Function Is Essential for Actinotrichia Alignment (Not Basal Keratinocytes)

**FIGURE 5:**
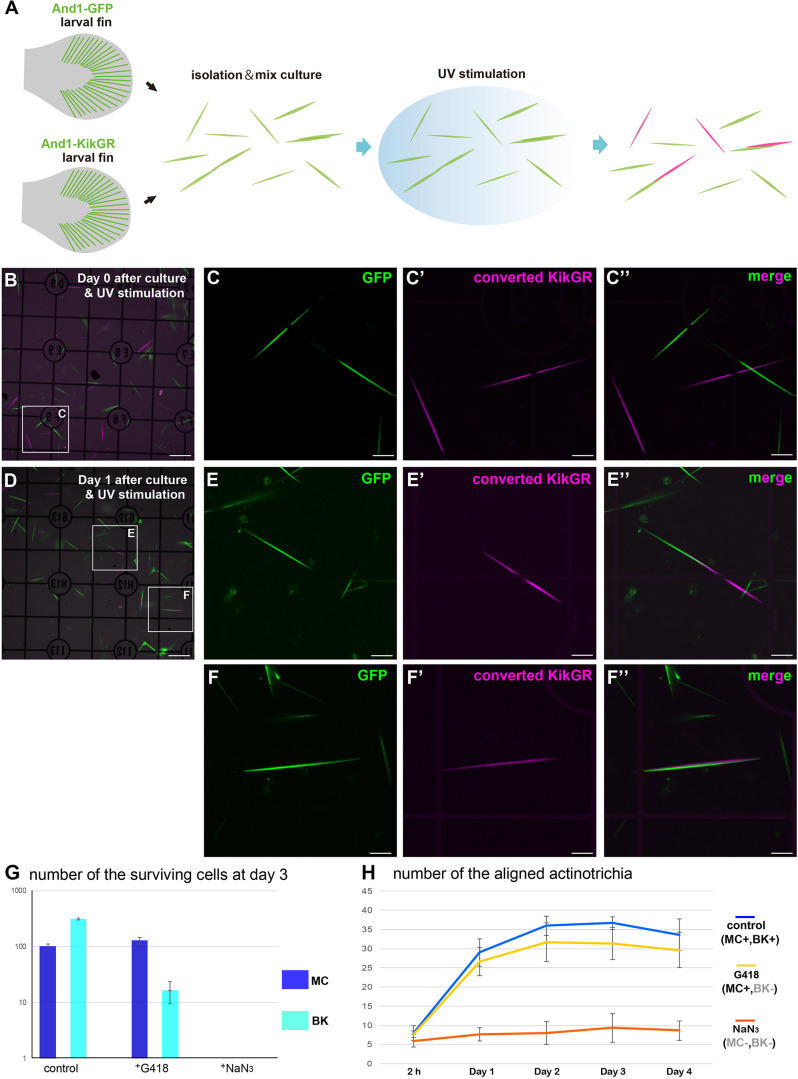
Mesenchymal cells are essential for the *in vitro* alignment of actinotrichia. **(A)** Illustration of the procedure for the *in vitro* mix culture experiment. Actinotrichia and cells were harvested from two different TG larval fins (TG; *and1* 1.4k: And1-GFP, *and1* 1.4k: And1-KikGR) and cultured on a Matrigel-coated dish. The isolated actinotrichia were stimulated by UV radiation *in vitro* and the aligned actinotrichia composed of fibrils labeled by two different fluorescent proteins were counted. **(B)** The fluorescence image at Day 0 (after 2 h of culture and UV stimulation). **(C–C′′)** Magnified images of the white box in **(B)**. **(D)** The fluorescence image at Day 1 after culture and UV stimulation. **(E–E′′)** Magnified images of the white box in **(D)**. Two actinotrichia were connected tip-to-tip and their orientation was aligned. **(F–F′′)** Magnified images of the white box in **(D)**. Two actinotrichia were connected with side-to-side and their orientation was aligned. **(G)** Number of the surviving mesenchymal cells and basal keratinocytes under the control, G418 (200 μg/ml)-treated condition and NaN_3_ (0.1%)-treated condition. **(H)** The aligned actinotrichia were increased in the G418-treated condition (only mesenchymal cells alive) but not in the NaN_3_-treated condition (cell-free state). MC, mesenchymal cell; BK, basal keratinocyte. Scale bars: 100 μm in **(B,D)**, 20 μm in **(C-C′′,E-E′′,F-F′′)**.

*In vivo*, actinotrichia are distributed as a sheet just below the basement membrane, holding onto mesenchymal cells (Figure 2). Actinotrichia production itself appears to be caused by mesenchymal cells and another cell type, basal keratinocytes; we have previously reported that basal keratinocytes also interact actively with actinotrichia (Kuroda et al., 2018). Therefore, we next decided to quantitatively investigate the effect of these cells in aligning the orientation of actinotrichia using a mixed-culture experimental system. Figure 5A shows the schematic diagram of the experiment. To label actinotrichia with two different fluorescent proteins, we used two TG zebrafish expressing And1-GFP and And1-KikGR (Supplementary Figure 3). We firstly established TG lines expressing And1-mCherry and And1-mRFP to label actinotrichia with red fluorescence. However, we were unable to observe specific fluorescence in the actinotrichia in these TG fish and instead observed a large number of vesicular fluorescence in the ECM (data not shown). We therefore decided to use photo-conversion using KikGR to visualize actinotrichia fibers with red fluorescence for this experiment. Actinotrichia were isolated from the fins of both fish and cultured along with the cells derived from the fins. The origin of each actinotrichia can be distinguished by fluorescence because KikGR turns red when exposed to UV after incubation (Supplementary Figure 3). We counted the number of actinotrichia, comprising two colors, green and red, as they were bundled *in vitro* after the culture. At day 0 (2 h), most actinotrichia were each oriented differently, even though they were close to each other (Figures 5B–C′′). On the contrary, after day 1, many aligned actinotrichia were observed (Figures 5D–F′′). In the case of bundled actinotrichia, the tips were aligned close together (Figures 5E–E′′) and the sides were aligned close together (Figures 5F–F′′ and Supplementary Movie 10). In controls, the number of bundled actinotrichia were increased between day 0 and day 4 (Figure 5H). Because mesenchymal cells and basal keratinocytes could not be completely separated, we used the drug (G418) to estimate the actinotrichia bundling activity of each cell. The survival of basal keratinocytes in the dish was sharply reduced by G418 treatment, whereas the mesenchymal cells were not reduced (Figure 5G and Supplementary Figure 4). This indicates that basal keratinocytes have little effect in the presence of G418. Moreover, both cells died quickly in the presence of NaN_3_ (Figure 5G and Supplementary Figure 4), and so, the effect of both cells could be completely eliminated under NaN_3_-treated conditions. Figure 5G shows the number of bundled actinotrichia for each condition. Under conditions in which only the mesenchymal cells could survive, the number of bundled actinotrichia was increased similar to the control (Figure 5H). Furthermore, no increase in bundle-forming actinotrichia was observed under conditions in which all cells were not viable (Figure 5H). These results indicate that the actinotrichia bundling observed *in vitro* is dependent on the activity of mesenchymal cells and not simply on the self-assembling property of collagen fibers.

### Inhibition of Actomyosin Activity Suppressed the Orientation Formation of Actinotrichia

**FIGURE 6:**
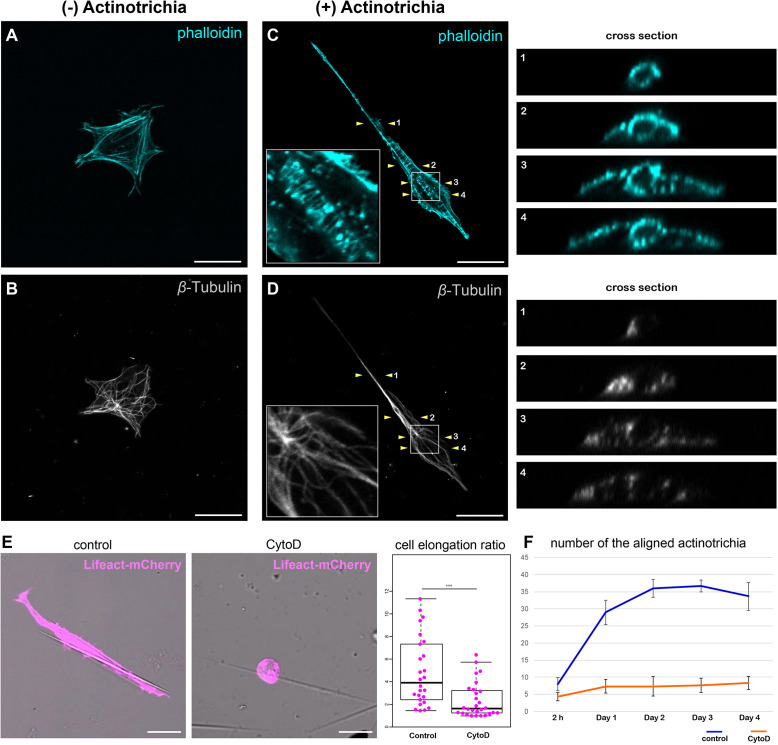
Inhibition of actin polymerization suppressed the orientation formation of actinotrichia. **(A,B)** A cultured mesenchymal cell without contact with actinotrichia was stained with phalloidin and anti-β-Tubulin antibodies. **(A)** Image of phalloidin staining and **(B)** anti-β-Tubulin antibody staining. Actin-rich fibers were detected below the plasma membrane and actin-rich filopodia structures were developed at the cell edge **(A)**. β-Tubulin localization was observed radially from the center of the cell **(B)**. **(C,D)** A cultured mesenchymal cell in contact with a single actinotrichia was stained with phalloidin and anti-β-Tubulin antibodies. The ortho-slice images between two yellow arrowheads are showed in the right panels and the magnified images of the white box are inset. **(C)** The image of phalloidin staining and **(D)** anti-β-Tubulin antibody staining. Strong accumulation of actin was detected around the actinotrichia. On the contrary, β-Tubulin localization was observed radially from the center of the cell and was not detected around the actinotrichia. **(E)** The morphology of the cultured mesenchymal cells at day 2 after culture under control and CytoD-treated conditions. The mesenchymal cells treated with CytoD tended to be unable to elongate along the actinotrichia axis. The elongation ratio of the CytoD treated cells is much lower than that of the control cells. **(F)** The number of aligned actinotrichia did not increased under the CytoD-treated condition. *P*-values: ***P < 0.001. Scale bars: 20 μm.

Mesenchymal cells have the property to elongate along the long axis of actinotrichia while holding the actinotrichia. Furthermore, mesenchymal cells can move actinotrichia and align their orientation. This behavior seems to require mechanical forces, suggesting cytoskeletal involvement. Therefore, we next observed the relationship between the mesenchymal cell cytoskeleton and actinotrichia to investigate the relationship between mesenchymal cell morphology and the cytoskeleton. First, in the absence of adhesion to actinotrichia, the actin skeleton of mesenchymal cells developed in a fibrous fashion along the inner surface of the cell membrane (Figure 6A). Actin-rich pseudopods were also observed at the edges of the cells (Figure 6A). In contrast, the microtubule skeleton developed radially from the center of the cell body, and this radial distribution of microtubule did not extend to the pseudopod (Figure 6B). Next, we observed the cytoskeleton of mesenchymal cells holding actinotrichia. Actin fibers were observed to be aligned perpendicular to the long axis of actinotrichia (Figure 6C). In cross-sections, actin fibers developed in a ring around the actinotrichia (see the cross section in Figure 6C). We also found that phospho FAK (pFAK) accumulated along the longitudinal axis of actinotrichia in conjunction with actin fibers (Supplementary Figure 5). FAK is a key factor of focal adhesions (FAs) which intermediate between the cellular actin network and ECM components like collagen complexes (Mitra et al., 2005; Martínez et al., 2020). This result strongly indicates that the actin cytoskeleton can support the physical interaction between the mesenchymal cells and actinotrichia. In contrast, the distribution of the microtubule skeleton does not appear to be significantly altered, despite the extreme deformation of the cells (Figure 6D). Microtubules were not observed around the actinotrichia, even in cross-sections (see the cross section in Figure 6D). These results suggest that the specific interaction between mesenchymal cells and actinotrichia is supported by the actin skeleton. Therefore, we next used CytoD to inhibit actin polymerization *in vitro* and examined the morphology of mesenchymal cells contacting the actinotrichia under these conditions. Inhibition of actin polymerization in mesenchymal cells suppressed their elongation along the long axis of actinotrichia (Figure 6E). In addition, the bundling of actinotrichia as observed in Figure 5 did not occur under the condition of CytoD treatment (Figure 6F). Cellular morphological changes caused by the rearrangement of actin cytoskeleton are regulated by Rho family GTPases such as RhoA (de Curtis and Meldolesi, 2012; Murali and Rajalingam, 2014). Therefore, we next examined the morphology of the cultured mesenchymal cells and the *in vitro* alignment of actinotrichia when Rho function was inhibited. As a result, the cell elongation ratio of the mesenchymal cells in contact with actinotrichia decreased when treated with Rho inhibitor CT04 (Supplementary Figure 6). In addition, there was no significant increase in the number of bundled actinotrichia as seen in control (Supplementary Figure 6). We next hypothesized that the source of the physical force is the contraction of actomyosin. Therefore, we next investigated the effect of Blebbistatin, a myosin II inhibitor. Interestingly, the cell elongation ratio decreased, and the bundling of actinotrichia did not occur under the condition of Blebbistatin treatment (Supplementary Figure 6). These results suggested that reorganization of actin regulated via Rho GTPase causes morphological changes in mesenchymal cells and promotes the formation of actinotrichia orientation, which may be driven by a myosin-dependent contraction force.

### Radial Elongation of Mesenchymal Cells via Actin Polymerization Is Essential for the Proper Orientation of Actinotrichia *in vivo*

**FIGURE 7:**
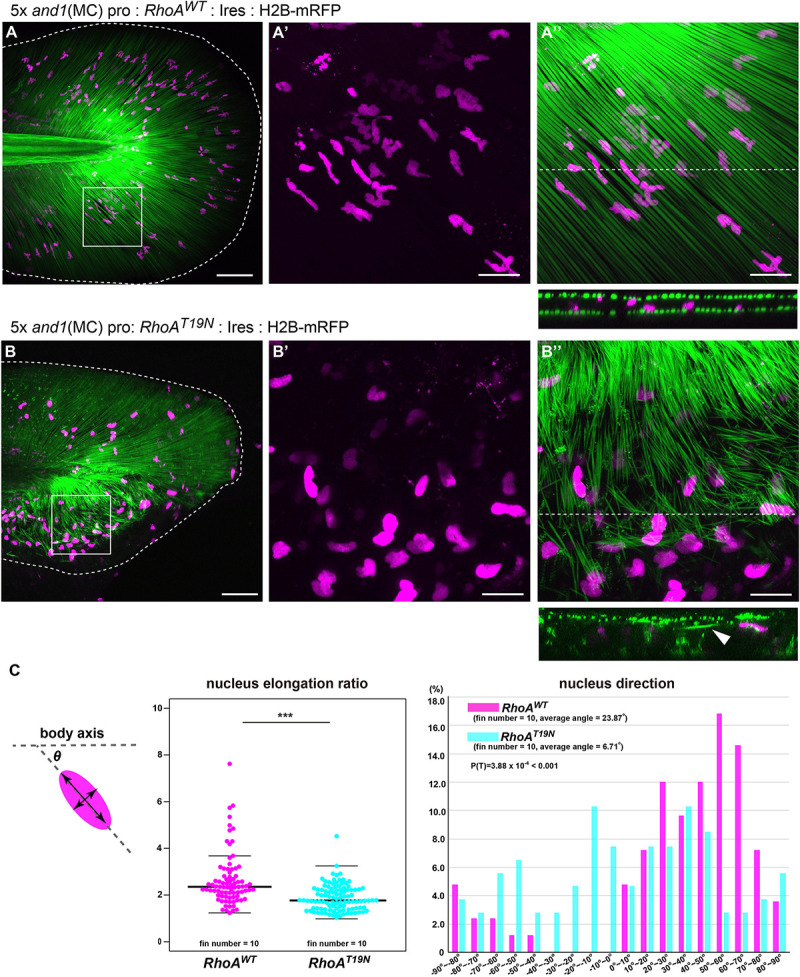
Suppression of actin polymerization in mesenchymal cells induced the collapse of the actinotrichia distribution *in vivo*. The distribution pattern of the actinotrichia and the nuclei of mesenchymal cells in the TG larva (3 dpf) expressing the wild-type *RhoA* **(A)** or dominant negative form of *RhoA* **(B)** in mesenchymal cells. The actinotrichia and nuclei of mesenchymal cells were visualized by And1^full^-GFP (green) and H2B-mRFP (magenta) respectively. **(A′,A′′)** The magnified images of the white box in **(A)**. **(A′)** Nuclei of mesenchymal cells and **(A′′)** nuclei of mesenchymal cells and actinotrichia are shown. The transverse section image at the white dot line in **(A′′)** is shown at the lower panel. **(B′,B′′)** The magnified images of the white box in **(B)**. **(B′)** Nuclei of mesenchymal cells and **(B′′)** nuclei of mesenchymal cells and actinotrichia are shown. The transverse section image at the white dot line in **(B′′)** is shown at the lower panel. Aberrant actinotrichia were distributed in a space between the two actinotrichia layers (white arrowhead). **(C)** The nucleus elongation ratio in mesenchymal cells expressing *RhoA*^DN^ is much lower than that in the control. The nucleus direction of mesenchymal cells in control larvae showed a radial distribution pattern, whereas, it was randomized in the larvae overexpressing *RhoA*^DN^. The nucleus of mesenchymal cells in the ventral-mid area of median fins were measured. *P*-values: ***P < 0.0001. Scale bars: 50 μm in **(A,B)**, 20 μm in **(A′,A′′,B′,B′′)**.

Next, to confirm the relationship between the actin skeleton and the actinotrichia orientation *in vivo*, we investigated the effect of mesenchymal cell-specific inhibition of actin polymerization on the orientation of actinotrichia. RhoA is one of a key regulator of the actin cytoskeleton and its protein structure and function are conserved in the various animals and tissues (Bhowmick et al., 2001; Zhang et al., 2012; Lam et al., 2015). When wild-type *RhoA* (*RhoA*^WT^) of zebrafish (control) was specifically expressed in the mesenchymal cells of fins, actinotrichia were normally ordered and radially aligned (Figures 7A,A′′). The nucleus of the mesenchymal cell was radially elongated, indicating that the cell is oriented along the actinotrichia (Figures 7A–A′′). In the *RhoA*^WT^ overexpression fins, many nuclei of the mesenchymal cells had a wavy shape (Figure 7A′). We confirmed by hoechst staining that the intact wild-type nuclei also have a similar wavy shape (data not shown), so it is considered that such a characteristic nucleus shape is normal. In contrast, in fish expressing a dominant negative form of RhoA (RhoA^T19N^) specifically in the mesenchymal cells, the orientation of actinotrichia was disrupted in the region of the fins where *RhoA*^T19N^ expression occurs (Figures 7B,B′′). In the larvae with strong expression, median fins were severely shrunk and we couldn’t evaluate the actinotrichia orientation. Therefore, we observed the phenotype of the F0 fish with mosaic expression. Cross-sectional observations showed that in control fins the actinotrichia were arranged in straight layers, whereas in *RhoA*^T19N^ overexpressed fins, abnormal wavy layers of actinotrichia were formed (see the cross section in Figures 7A,B). We also found that aberrant actinotrichia distributed in a space between the two actinotrichia layers were also observed in *RhoA*^T19N^ overexpressed fins (see the cross section in Figure 7B). In addition, the nuclei of mesenchymal cells had a reduced elongation ratio and disordered directional regularity (Figures 7A′,B′,C). Furthermore, *RhoA*^T19N^ overexpressed fish showed the defect in the morphology of median fin folds (Supplementary Figure 7). These results indicate that the actin cytoskeleton supports the interaction between mesenchymal cells and actinotrichia *in vivo*, leading to the proper orientation of the actinotrichia.

## Discussion

In this study, we describe the mechanism by which actinotrichia align at the zebrafish fins. First, 3D images of the fin tip obtained using FIB-SEM showed that the pseudopodia of mesenchymal cells encompassed multiple actinotrichia. Next, we observed how purified mesenchymal cells bundled actinotrichia using pseudopodia in an *in vitro* culture system. Furthermore, inhibition of actin polymerization which drives pseudopodia formation and cell morphological changes in mesenchymal cells disrupted the orientation of actinotrichia in the larval fins. This strongly suggests that the behavior of mesenchymal cells observed in the *in vitro* system is related to the phenomenon of actinotrichia alignment within the fins. This was also the first time that the cells were observed to bundle collagen fibers derived from tissues in real time.

There are other examples of *in vitro* studies on the relationship between cells and ECM proteins. Jansen et al. (2013) cultured human fibroblasts using a fibrin fiber gel and found that the network of fibrin fibers was changed dramatically with morphological changes in fibroblasts and that the stiffness of the fibrin gel was increased (Jansen et al., 2013). Xie et al. (2017) performed experiments on human mesenchymal cells using a polymerized collagen gel and reported reorganization of the network of collagen fibers due to the action of the cells. In their experiments, the orientation pattern of the collagen fibers was reorganized in the direction of the cell protrusions due to the tension emitted by the mesenchymal cells, which also increased the stiffness around the cells (Xie et al., 2017). Moreover, this reorganization of the collagen network was caused by inhibition of cell contraction by actin and myosin (Xie et al., 2017). Although these experiments clearly demonstrate the contribution of cells to collagen complex formation, the individual collagen fibers are too thin to directly observe the interaction with cells. On the contrary, although actinotrichia are a somewhat unique collagen fiber, their characteristic shape and size allow us to observe the physical interaction between cells. In present study, we found the inhibition of myosin II *in vitro* suppressed the morphological changes of mesenchymal cells that bundle actinotrichia in the normal condition. This result suggests that the contractile force of actomyosin plays an important role in the orientation formation of actinotrichia, and the underlying mechanism may be common with the orientation formation of collagen in other tissues.

In this study, we found that mesenchymal cells use their pseudopodia to align actinotrichia; however, the FIB-SEM 3D images suggest that the pseudopodia of mesenchymal cells have another function of regulating the fusion of actinotrichia. In general, collagen fibers grow large through repeated fusions due to their self-assembly properties (Graham et al., 2000; Kadler et al., 2000; Holmes et al., 2010). Actinotrichia are thinner and denser at the fin tip, but gradually become thicker and less dense from the tip of the fin to its base (Kuroda et al., 2018). This suggests that the actinotrichia are gradually fused from the fin tip toward the root. The degree of fusion must be controlled precisely, as the thickness of the actinotrichia is kept almost constant at each level. In the FIB-SEM 3D images, the pseudopodia of mesenchymal cells invade the actinotrichia interstices and prevent neighboring actinotrichia from physically contacting each other. If this state is maintained, fusion of actinotrichia should be prevented. In addition, retracting the pseudopodia would bring them into direct contact with the actinotrichia, thus facilitating fusion. In other words, the pseudopodia may physically regulate actinotrichia fusion by suppressing actinotrichia contact. It is thus important to investigate the pseudopodia of mesenchymal cells in the amino acid substitution mutant of *col1a1a*, because it has been observed that the amino acid substitution mutation of *col1a1a* results in thicker actinotrichia (Fisher et al., 2003; Durán et al., 2011). The relationship between the pseudopodia of cells and the fusion of collagen fibrils has been similarly indicated in previous studies (Canty et al., 2006; Kalson et al., 2015). Collagen fibers of the tendons in mice are known to undergo fusion between stage E15.5 and 6 weeks of age, with a rapid increase in the thickness of the fibers. Tenocytes, a type of fibroblasts, are distributed in the crevices of collagen bundles, extending their long filopodia and encasing the collagen bundles. As the length and elongation direction of the filopodia changes with development, Kalson et al. (2015) suggested that the filopodia of tenocytes may regulate the fusion of collagen fibrils. The relationship between tenocytes and collagen fibrils is very similar to that of actinotrichia with the fin mesenchymal cells, suggesting that there may be a commonality in the regulation of collagen polymer formation. Our FIB-SEM 3D data showed that mesenchymal cells develop multiple membrane protrusions that are intricately intertwined with actinotrichia. The membrane protrusions also played a central role in the manipulation of actinotrichia *in vitro*. Therefore, it should be important to understand how the pseudopods formation is controlled in mesenchymal cells. Many studies have shown that the formation of pseudopods is regulated by small GTPases such as CDC42 and Rac1 (de Curtis and Meldolesi, 2012; Murali and Rajalingam, 2014). Therefore, in order to analyze the interaction between mesenchymal cells and actinotrichia in more detail, it is important to manipulate CDC42 and Rac1 in specific cells.

The cellular morphology and the direction of cell migration depends on properties such as the density and stiffness of the surrounding ECM components (Yeung et al., 2005; Wehner et al., 2013; Charrier et al., 2018; Matellan and del Rıó Hernández, 2019). In fins, mesenchymal cells are mostly shaped rounded at the base region and are elongated at the fin tip region. Actinotrichia, on the other hand, are thicker at the base of fin and very thin at the fin tip (Kuroda et al., 2018). Our *in vitro* observation showed that mesenchymal cells become very thin when they wrap around the thin tip of actinotrichia, suggesting that the regional differences of mesenchymal cells morphology depends on the thickness of the actinotrichia.

The strong adhesion of mesenchymal cells to actinotrichia also indicates that actinotrichia can also affect the migration of mesenchymal cells. Our *in vitro* observations showed that pFAK accumulated at the attachment point to actinotrichia and F-actin was also recruited. This suggests that this adhesion occurs via Integlin on the membrane of mesenchymal cells and the polymerization of F-actin is facilitated by RhoA. To clarify the role of RhoA in this system should be one of the key issue to understand the mechanism of fin growth.

Previous studies suggest that Vimentin, intermediate filament, is highly expressed in mesenchymal cells of zebrafish fin and is involved in wound repair (LeBert et al., 2018). The specific functions of Vimentin in mesenchymal cells are not understood and the involvement of Vimentin in interaction with actinotrichia is unknown. Recently, it has been reported that Vimentin promotes collective migration by regulating the localization of the actomyosin network and focal adhesions in primary culture experiments using rat astrocytes (De Pascalis et al., 2018). Vimentin may function in the interaction between mesenchymal cells and actinotrichia by regulating the reorganization of the actin skeleton and the localization of the focal adhesion. In the future, we need to visualize Vimentin itself and observe the dynamics of Vimentin and the actin skeleton during the interaction between mesenchymal cells and actinotrichia by live imaging.

Actinotrichia are a key structure in the formation of fins. They are formed at the tip of the fin, aligning, fusing, and thickening as the fin grows, forming a scaffold for bone formation, and eventually break down and disappear. To understand the morphogenesis of fins, cells involved in each task need to be identified along with their mechanism. In a previous study, we showed that basal keratinocytes can solely form actinotrichia in the cell (Kuroda et al., 2018). In addition, the present study suggests that mesenchymal cells regulate actinotrichia alignment and fusion. Although the dynamics of actinotrichia are still unknown at this point, we believe that direct observation of cell-actinotrichia interactions in an *in vitro* system will continue to be an effective method of research in the future.

## Data Availability Statement

All datasets generated for this study are included in the article/Supplementary Material.

## Ethics Statement

The animal study was reviewed and approved by Osaka University.

## Author Contributions

All authors contributed to this manuscript. JK, TI, AHI, and SK designed the study and interpreted the data. JK, TI, and TA performed the experiments. JK, TI, and AHI analyzed the data. JK, TI, AHI, and SK drafted the manuscript.

## Conflict of Interest

The authors declare that the research was conducted in the absence of any commercial or financial relationships that could be construed as a potential conflict of interest.
