# Supplementary material for: The Physical Role of Mesenchymal Cells Driven by the Actin Cytoskeleton Is Essential for the Orientation of Collagen Fibrils in Zebrafish Fins
Source: Front Cell Dev Biol. 2020 Oct 14;8:580520. doi: 10.3389/fcell.2020.580520 (PMC7591588; doi:10.3389/fcell.2020.580520)
Supplement: Supplementary file 1 [file Data_Sheet_1.docx]

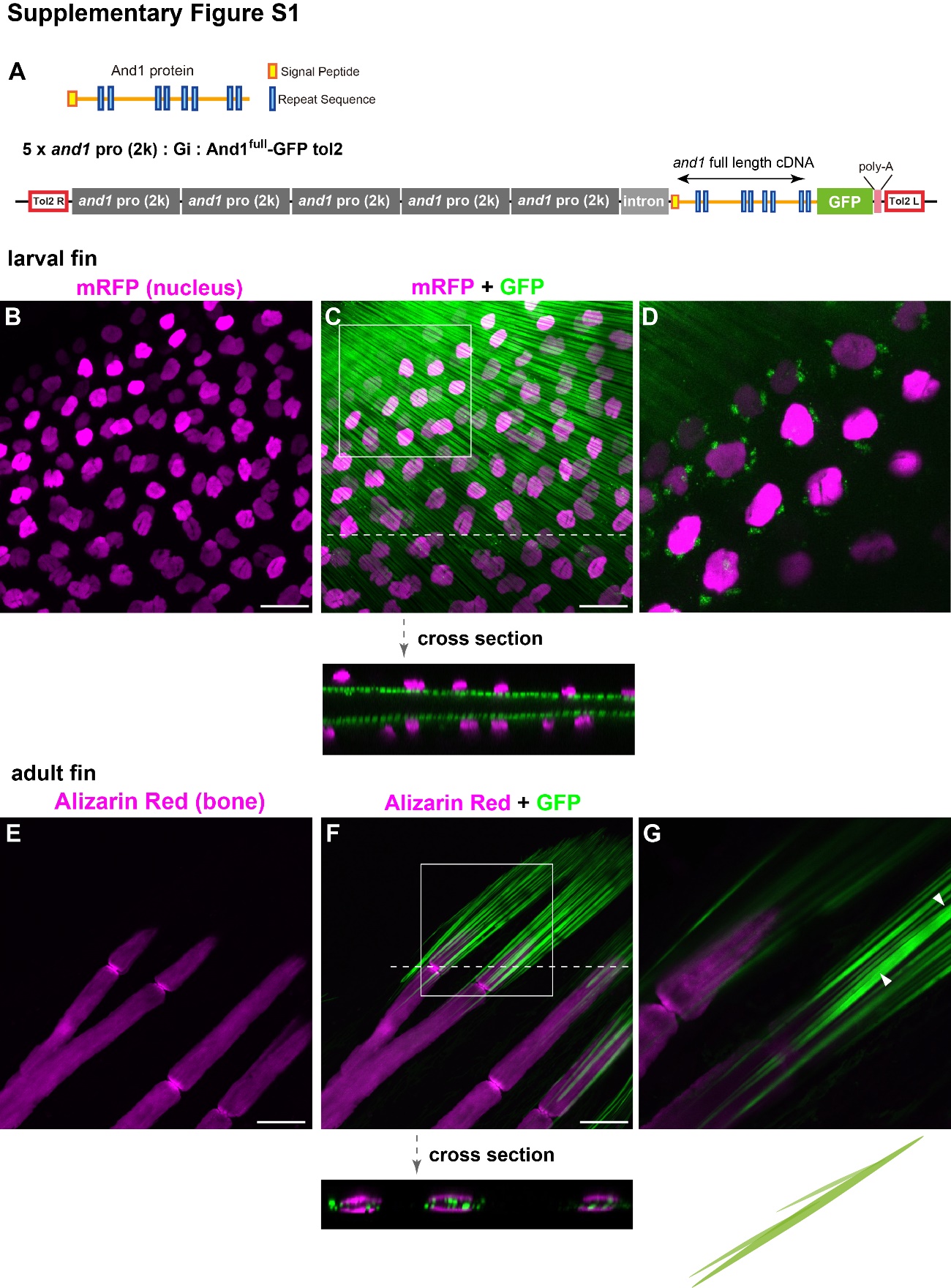


**Supplementary Figure S1**

Actinotrichia visualization by the overexpression of full length And1-tagged GFP under the 5x*and1*(2k) promoter.

(A) Schematic illustration of the zebrafish And1 protein and the tol2 plasmid construct for actinotrichia visualization. Five 2k *and1* promoters (Lalonde et al., 2016) are connected in tandem upstream of Globin intron. (B) The nucleus of basal keratinocytes at the larval fin fold in TG: *and1*(1.4k pro):H2B-mRFP. (C) The merged image of mRFP and And1^full^-GFP. The cross-section image at the white dotted line is shown in the lower panel. Each actinotrichia is clearly visualized and they normally form two straight layers under the basal keratinocyte sheets. (D) The magnified image of the white box in (C). Several small dot-like accumulations of GFP are observed around each nucleus. (E) Fin rays in an adult caudal fin stained with Alizarin red. Each fin ray forms regular joint structures and branches normally around the fin tip. (F) The merged image of Alizarin red staining and And1^full^-GFP. The cross-section image at the white dotted line is shown in the lower panel. Each actinotrichia is oriented straight from the inside of the fin rays to the fin tip. (G) The magnified image of the white box in (F). Three thinner actinotrichia fibrils fused with each other and formed a thicker bundle. Scale bars: 20μm in B and C, 50μm in E and F.


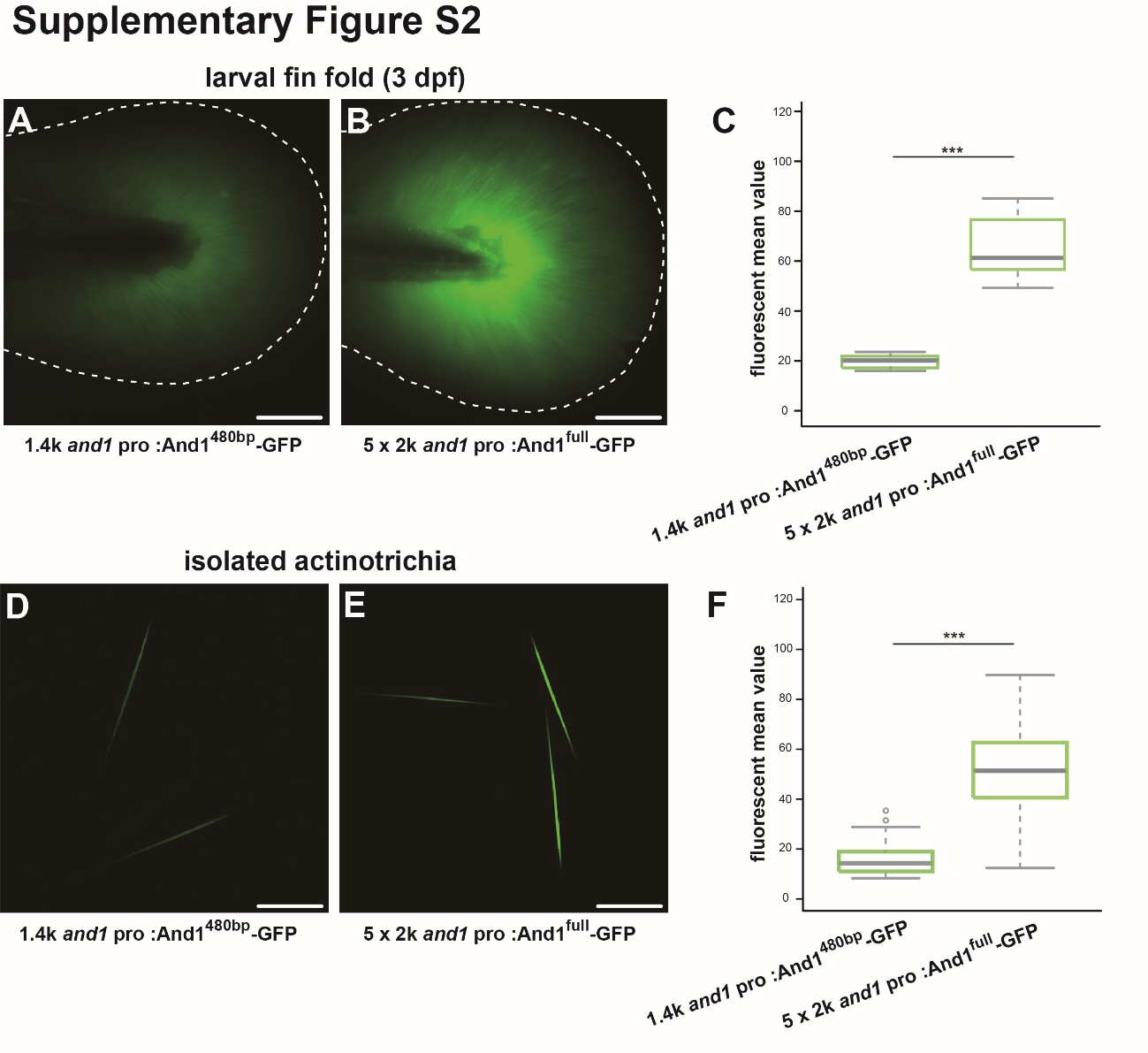


**Supplementary Figure S2**

Fluorescence level of the actinotrichia visualized by GFP labeling.

Fluorescence images of the larval fin fold at 3 dpf in (A) Tg (1.4k *and1* pro: And1^480bp^-GFP) and (B) Tg (5x2k *and1* pro: And1^full^-GFP). (C) The GFP fluorescent level in Tg (5x2k *and1* pro: And1^full^-GFP) is about three times higher than that in Tg (1.4k *and1* pro: And1^480bp^-GFP). Fluorescence images of the isolated actinotrichia from (D) Tg (1.4k *and1* pro: And1^480bp^-GFP) and (E) Tg (5x2k *and1* pro: And1^full^-GFP). (F) The GFP fluorescence level in Tg (5x2k *and1* pro: And1^full^-GFP) is about three times higher than that in Tg (1.4k *and1* pro: And1^480bp^-GFP). P-values: *** P *<* 0.0001. Scale bars: 100μm in A and B, 50μm in D and E.


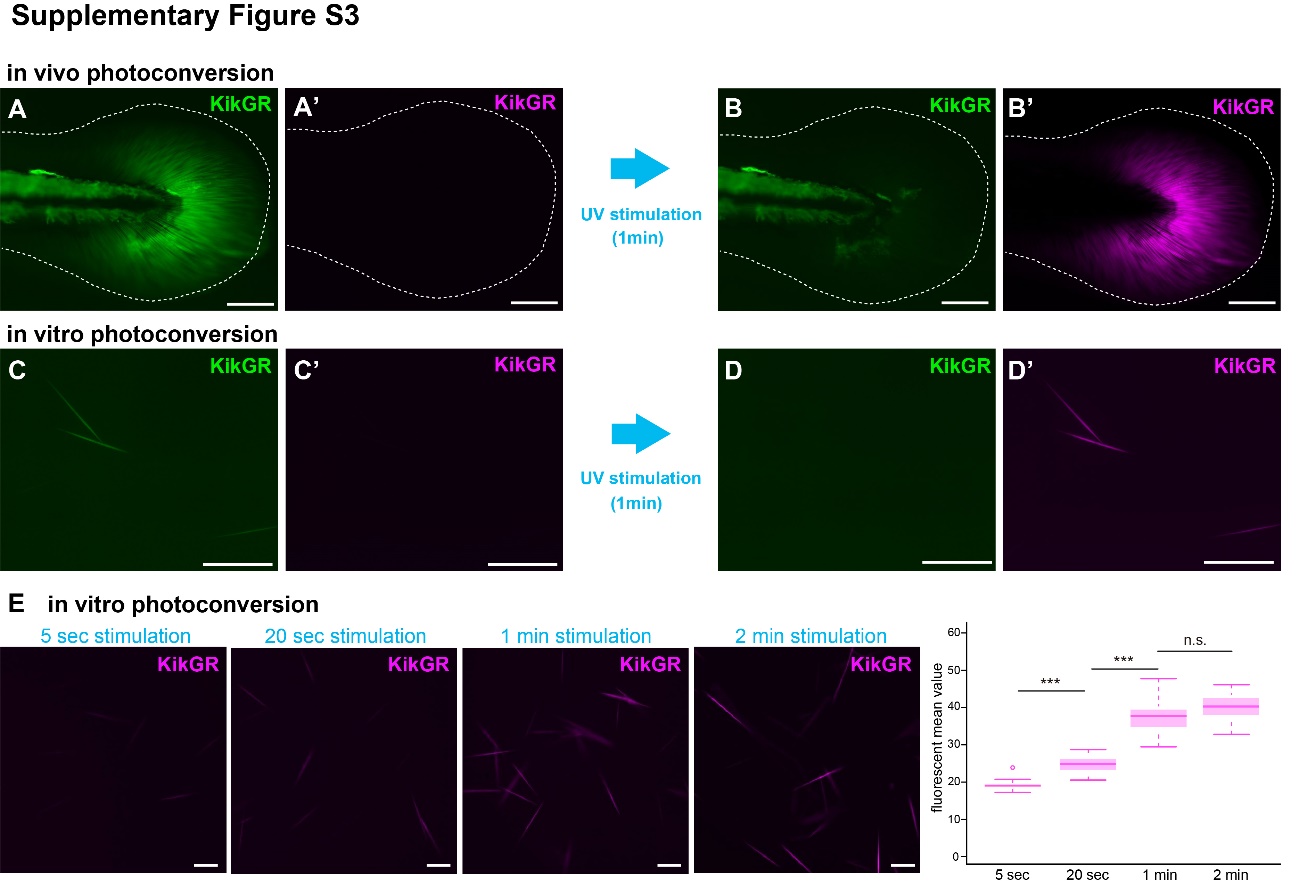


**Supplementary Figure S3**

Photo-conversion of actinotrichia by UV stimulation.

(A-B’) The TG larval fin at 3dpf expressing And1-KikGR under the *and1* (1.4k) promoter. (A) Actinotrichia in the fin had green fluorescence before UV stimulation and (A’) no red fluorescence was observed. (B) The green fluorescence of actinotrichia disappeared after UV stimulation and (B’) strong red fluorescence was observed. (C-D’) Isolated actinotrichia from the TG larval fins at 3dpf expressing And1-KikGR under the *and1* (1.4k) promoter. (C) Actinotrichia in a culture dish had green fluorescence before UV stimulation and (C’) no red fluorescence was observed. (D) The green fluorescence of actinotrichia is disappeared after UV stimulation and (D’) strong red fluorescence was observed. (E) Red fluorescence intensity of the photo-converted actinotrichia increased after 5 sec to 1 min of UV stimulation. There was no difference in the intensity between 1 min and 2 min of stimulation. The measured mean value of each intensity is shown in the right graph. Scale bars: 100μm in A – B’, 50μm in C – E. P-values: *** P *<* 0.0001.


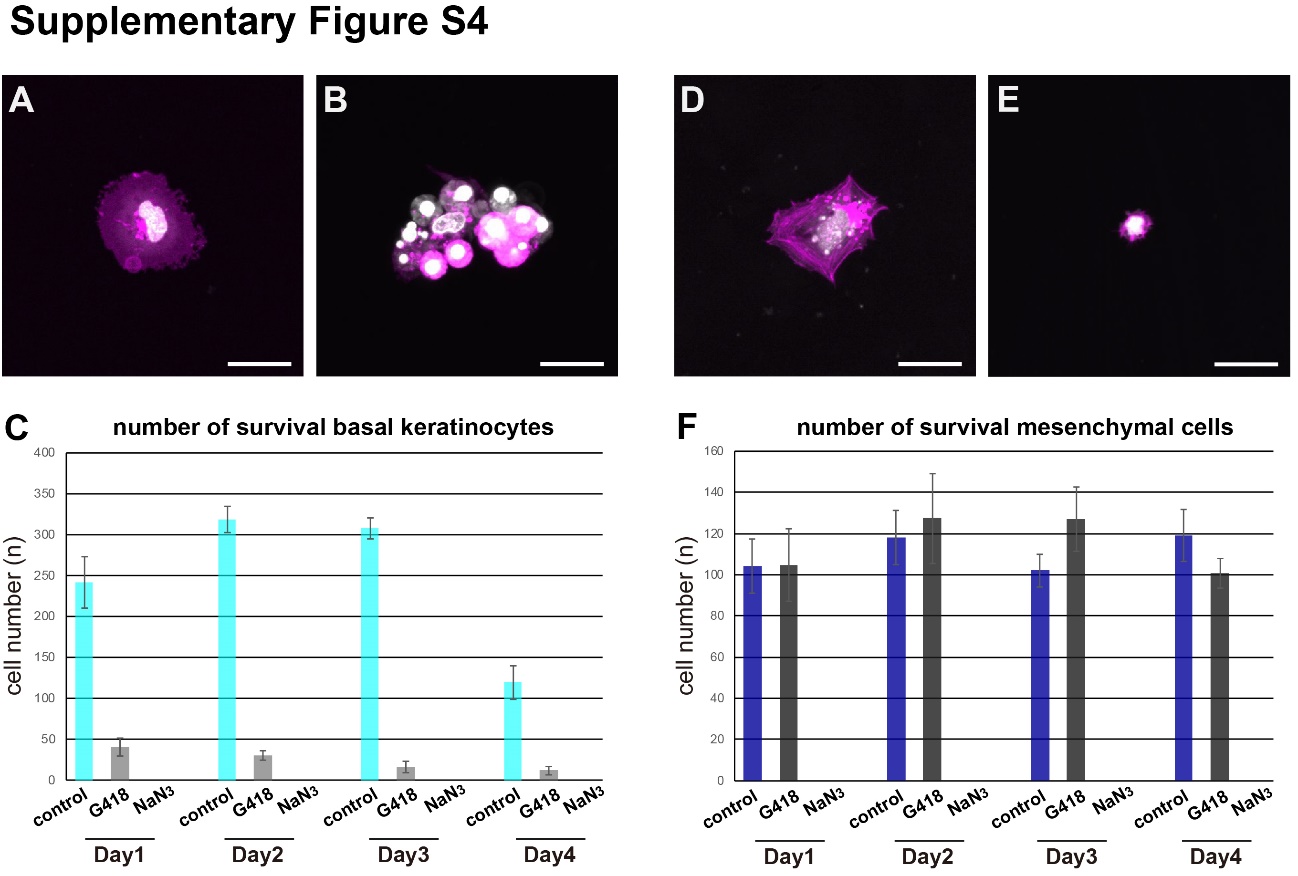


**Supplementary Figure S4**

Number of surviving cells cultured in the G418 or NaN_3_ treatment condition.

The basal keratinocytes and mesenchymal cells were isolated from the fins of the reporter TG fish and were cultured on a Matrigel-coated dish. (A and B) The cultured basal keratinocytes expressing mCherry-CaaX (magenta). The nuclei were labelled with Syto9 (white). (D and E) The cultured mesenchymal cells expressing Lifeact-mCherry (magenta). The nuclei were labelled with Syto9 (white). The surviving cells were well spread and adhered to the dish (A and D). In contrast, died cells shrank and were detached from the dish (B and E). (C) The number of surviving basal keratinocytes was dramatically decreased under the G418 (200μg/ml) treatment condition compared to the control. There were no surviving basal keratinocytes in the NaN_3_ (0.1%) treatment condition. (F) G418 (200μg/ml) treatment did not affect the surviving mesenchymal cells at all. There were no surviving mesenchymal cells in the NaN_3_ (0.1%) treatment condition. Scale bars: 20μm.

**
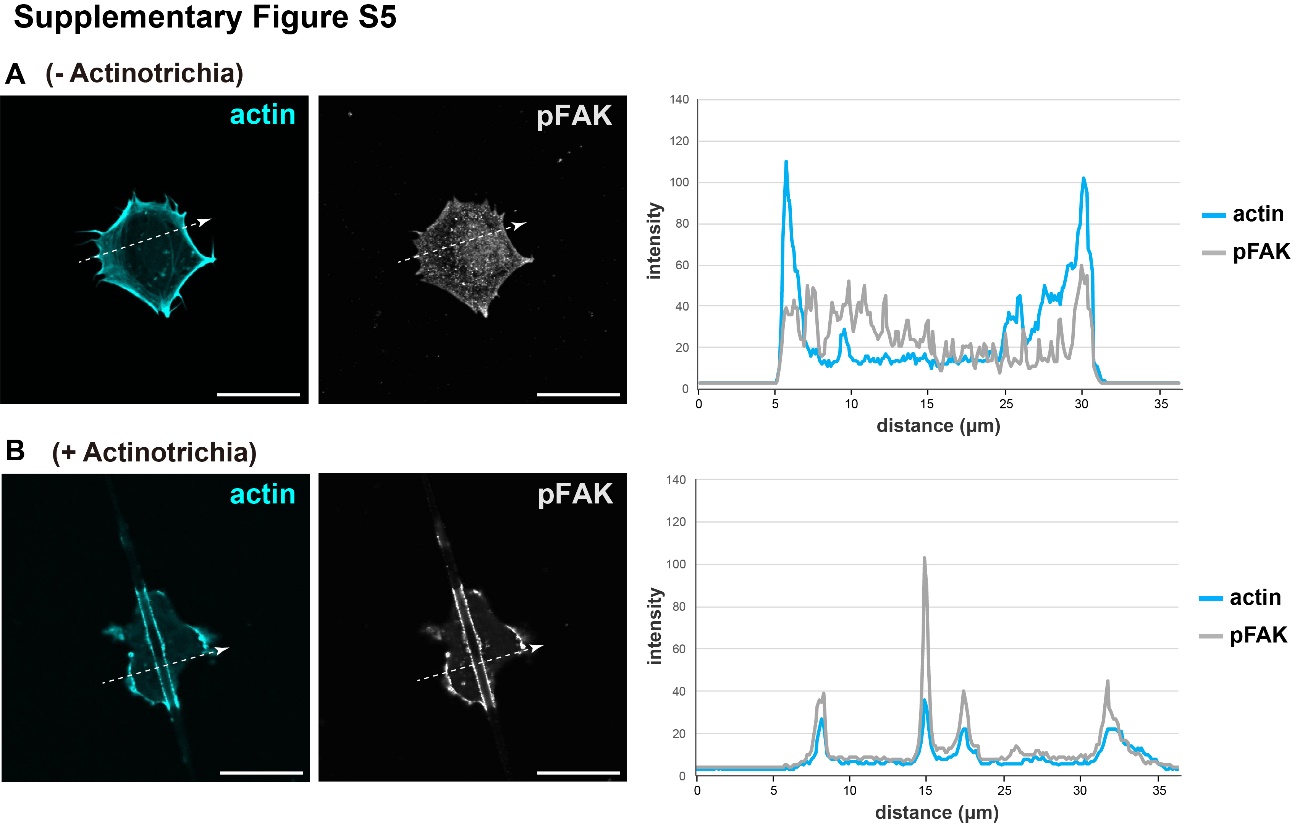
**

**Supplementary Figure S5**

The localization pattern of pFAK in cultured mesenchymal cells holding the actinotrichia.

The mesenchymal cells cultured on a Matrigel-coated dish were stained with phalloidin and anti-pFAK antibodies. (A) A mesenchymal cell not in contact with actinotrichia. Actin strongly accumulated around the cell margin, whereas pFAK was uniformly distributed in basal area of the cell. The fluorescence intensity of actin and pFAK staining are shown in the right graph. (B) A mesenchymal cell attaching to a single actinotrichia. Strong accumulation of actin and pFAK was observed along the longitudinal axis of the actinotrichia. The fluorescent intensity of actin and pFAK staining are shown in the right graph. The intensity of the two waves increased around the actinotrichia region (distance around 15 to 20 μm). Scale bars: 20μm.


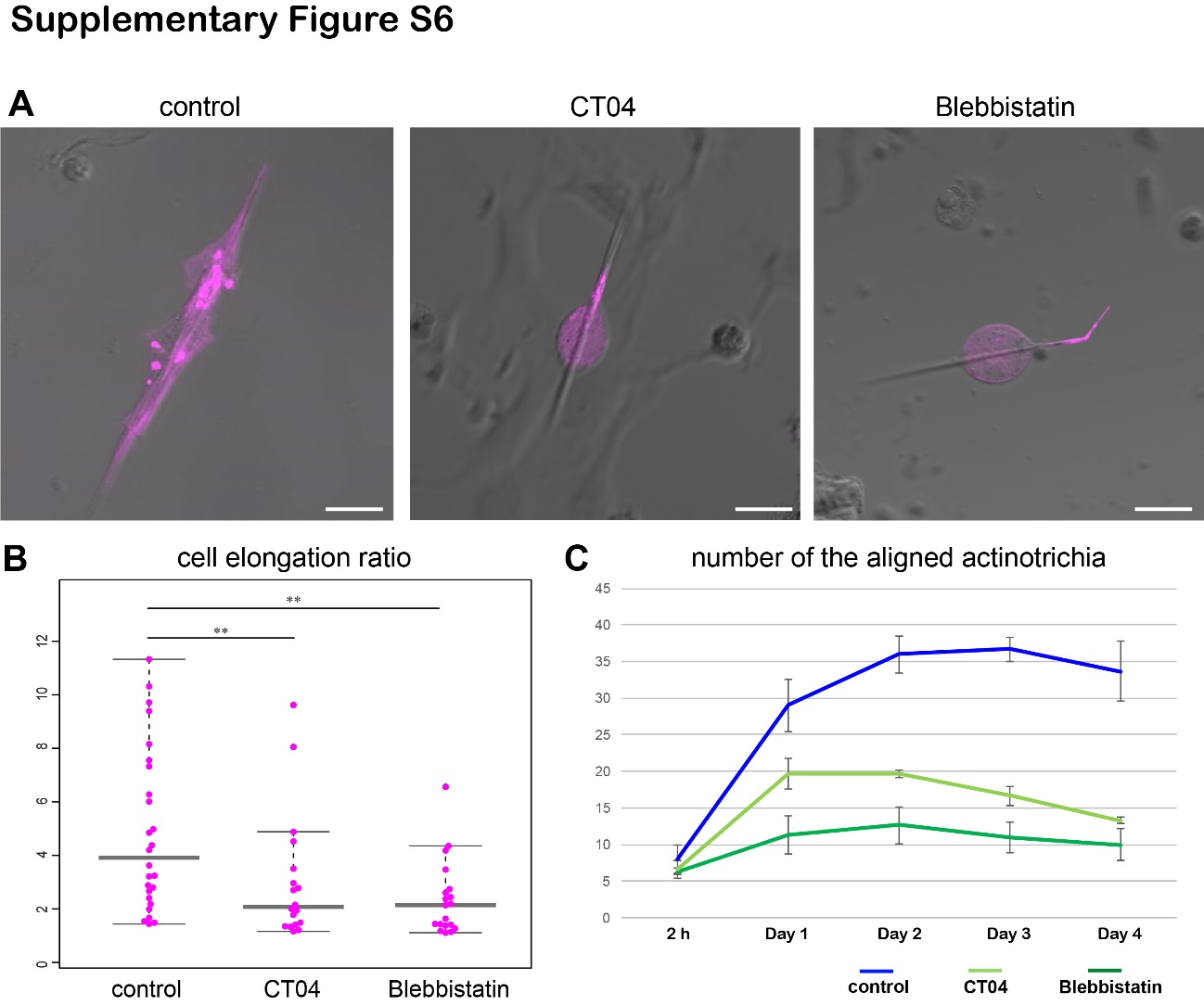


**Supplementary Figure S6**

Actomyosin activity of mesenchymal cells is required for the orientation formation of actinotrichia.

(A) The morphology of cultured mesenchymal cells in contact with a single actinotrichia on a Matrigel-coated dish. Mesenchymal cells were isolated from TG larval fins (TG; 5ˣ*and1*(MC): Lifeact-mCherry) and treated with 0.1% DMSO (control), Rho inhibitor (CT04) and Blebbistatin. (B) The cell elongation ratio of the mesenchymal cells at day 2 after culture under control and inhibitor-treated conditions. (C) Number of the aligned actinotrichia was increased in the control condition but not in the CT04-treated and Blebbistatin-treated condition. P-values: ** P *<* 0.001. Scale bars: 20μm.


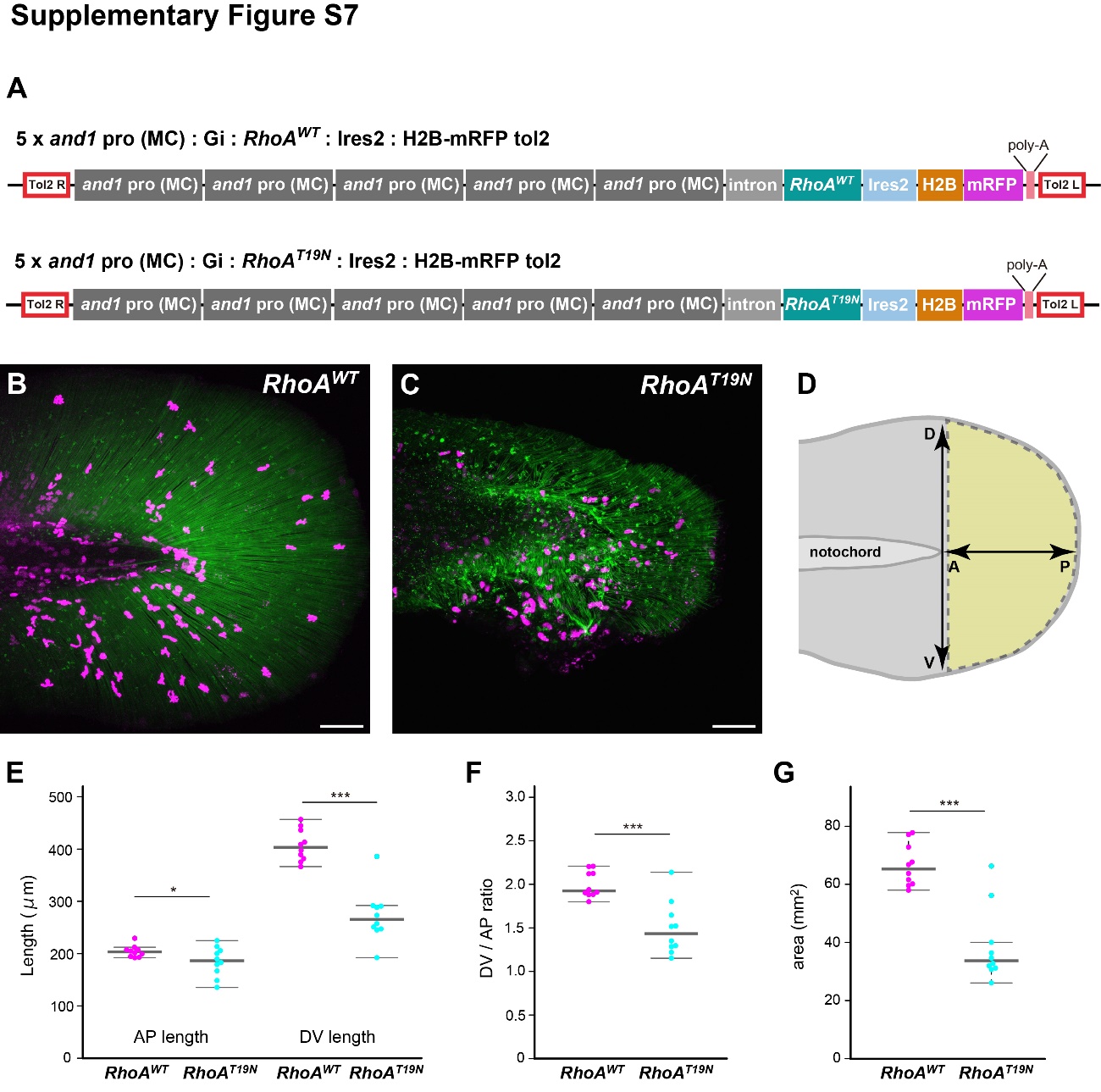


**Supplementary Figure S7**

Suppression of actin polymerization in mesenchymal cells induced the median fins malformation.

(A) Schematic illustration of the tol2 plasmid construct for the overexpression of wild type *RhoA* (*RhoA^WT^*) or dominant negative form of *RhoA* (*RhoA^T19N^*). (B and C) Confocal images of the median fins of 3 dpf larvae. (B) *RhoA^WT^* overexpressed median fin and (C) *RhoA^T19N^* overexpressed median fin. The actinotrichia and the nuclei of mesenchymal cells were visualized by And1^full^-GFP and H2B-mRFP respectively. (D) Schematic of median fin fold measurements. (E) AP length and DV length, (F) DV/AP ratio and (G) fin area (shown in yellow area in D) are measured. P-values: * P < 0.05, *** P *<* 0.0001. A, anterior; P, posterior; D, dorsal; V, ventral. Scale bars: 50μm.

**Additional files**

**Supplementary Movies**

Supplementary Movie 1. Serial 430 SEM images of xy plane in the tip of a median fin

fold at 3 dpf larva. 12.9 x 8.0 x 6.5 μm^3^ region with 8 x 10 x 15 nm^3^ voxel.

Supplementary Movie 2. Serial 759 SEM images of xz plane in the tip of a median fin

fold at 3 dpf larva. 12.9 x 8.0 x 6.5 μm^3^ region with 8 x 10 x 15 nm^3^ voxel.

Supplementary Movie 3. Serial 1556 SEM images of yz plane in the tip of a median fin

fold at 3 dpf larva. 12.9 x 8.0 x 6.5 μm^3^ region with 8 x 10 x 15 nm^3^ voxel.

Supplementary Movie 4. Result of consecutive slices from 3 directions of Serial Block

Face with coverlaid actinotrichia and mesenchymal cell models. 12.9 x 8.0 x 6.5 μm^3^

region with 8 x 10 x 15 nm^3^ voxel.

Supplementary Movie 5. 3D rotation movie of the fin mesenchymal cell interacting

with actinotrichia. The mesenchymal cell was labelled with Lifeact-mCherry and

actinotrichia were labelled with And1^full^-GFP. The cell and actinotrichia were cultured on

Matrigel-coated dish. z: 9.9 µm. Scale bar: 5 μm.

Supplementary Movie 6. 3D rotation movie of the fin mesenchymal cell interacting

with actinotrichia. The mesenchymal cell was labelled with Lifeact-mCherry and

actinotrichia were labelled with And1^full^-GFP. The cell and actinotrichia were

cultured on type1 collagen-coated dish. z: 7.7 µm. Scale bar: 10 μm.

Supplementary Movie 7. 3D rotation movie of the fin mesenchymal cell interacting

with actinotrichia. The mesenchymal cell was labelled with Lifeact-mCherry and

actinotrichia were labelled with And1^full^-GFP. The cell and actinotrichia were

cultured on type4 collagen-coated dish. z: 8.0 µm. Scale bar: 10 μm.

Supplementary Movie 8. Live imaging of the single mesenchymal cell interacting with

the two actinotrichia fibrils. Mesenchymal cells and actinotrichia fibrils were visualized

with Lifeact-mCherry and An1^full^-GFP, respectively. The cells and actinotrichia were

cultured on the Matrigel-coated dish. Each confocal images were scanned at 6min × 110

times. GaAsP detector and 488 nm, 561 nm laser were used for imaging. z: 16 µm.

Scale bar: 10 μm.

Supplementary Movie 9. Live imaging of the two mesenchymal cells interacting with

the three actinotrichia fibrils. Mesenchymal cells and actinotrichia fibrils were

visualized with Lifeact-mCherry and An1^full^-GFP, respectively. The cells and

actinotrichia were cultured on the Matrigel-coated dish. Each confocal images were

scanned at 6min × 96 times. GaAsP detector and 488 nm, 561 nm laser were used for

imaging. z: 19 µm. Scale bar: 10 μm.

Supplementary Movie 10. 3D rotation movie of the aligned actinotrichia fibrils.

Four fibrils visualized And1-GFP and photoconverted-And1-KikGR were physically

connected and aligned side by side. The cells and actinotrichia derived from the fins of

TG larvae were cultured on the Matrigel-coated dish. z: 8 µm. Scale bar: 10 μm.
